# Baseline Analysis of Endophytic Fungal Associates of *Solenopsis invicta* Buren from Mounds across Five Counties of Guangdong Province, China

**DOI:** 10.3390/jof9030377

**Published:** 2023-03-20

**Authors:** Bamisope Steve Bamisile, Junaid Ali Siddiqui, Lei Nie, Atif Idrees, Luis Carlos Ramos Aguila, Chunsheng Jia, Yijuan Xu

**Affiliations:** 1 Department of Entomology, South China Agricultural University, Guangzhou 510642, China; nielei98@stu.scau.edu.cn; 2 Henry Fok School of Biology and Agriculture, Shaoguan University, Shaoguan 512005, China; chunshengjia@sina.com; 3 College of Agriculture, College of Tobacco Science, Guizhou University, Guiyang 550025, China; junaidali206@gzu.edu.cn; 4 Guizhou Provincial Key Laboratory for Agricultural Pest Management of the Mountainous Region, Scientific Observing and Experimental Station of Crop Pest in Guiyang, Ministry of Agriculture, Institute of Entomology, Guizhou University, Guiyang 550025, China; atif_entomologist@yahoo.com; 5 Key Laboratory of Vegetation Restoration and Management of Degraded Ecosystems, South China Botanical Garden, Chinese Academy of Sciences, Guangzhou 510650, China; luiscarlos@scbg.ac.cn

**Keywords:** alien species, external symbionts, microbial communities, RIFA, endophytic fungi-insect host interaction, social insects

## Abstract

Red imported fire ants mounds have been suggested as a potential reservoir for beneficial entomopathogenic fungal species that are vital for more complex roles in the ecosystem aside from infecting the insects. In the current study, the assemblage of fungal symbionts of the red imported fire ants (RIFA) were obtained across five cities in Guangdong Province, China. The sampling areas were selected because of high occurrence of fire ants mounds in the regions. Mound soils, plant debris within mounds, and ants were collected from three sampling locations in each city for potential isolation of entomopathogenic fungal associates of RIFA. All samples were collected during the spring of 2021. Following successful isolation from substrates, the patterns of fungal species composition, and richness were evaluated. In total, 843 isolates were recovered, and based on their phenotypic distinctiveness and molecular characterization based on DNA sequences of multiple loci including the ITS, SSU, and LSU regions, 46 fungal taxa were obtained, including 12 that were unidentified. Species richness and abundance was highest in the mound soils, while the lowest value was recorded from the ant body. As per the different locations, the highest abundance level was recorded in Zhuhai, where 15 fungal taxa were cultivated. The most common taxa across all substrates and locations was *Talaromyces diversus*. A baseline analysis of the fungal community composition of RIFA would better our understanding on the interactions between these social ants and their associated microbial organisms, and this knowledge in turn would be important for the successful management of the RIFA.

## 1. Introduction

The microbial communities inhabiting the soil have rich diversity, and their distribution is based on the soil type, climatic conditions, and soil use (i.e., whether the soil is used for agricultural purposes or not) [1]. According to available data, a potential 1.5 million fungal species are contained in the soil, while only about 10% of these abundant microorganisms have been studied until recently [2]. The examined species include entomopathogenic, endophytic, saprophytic, and some edible fungi [3]. For the entomopathogenic species, approximately 90 genera and over 700 species have been reported so far [4,5,6].

Red imported fire ants, *Solenopsis invicta* Buren (Hymenoptera: Formicidae), are difficult to control due to their aggressiveness, effective foraging, and their ability to mobilize rapidly and actively attack intruders when their mounds are disturbed [7]. These social ants are notorious for invading other exotic and native ant species, which could eventually result in the displacement or elimination of essential native species. It is therefore important to keep a close watch on the activities of these social insects, especially monitoring their rate of dispersal and evaluating the influence of environmental conditions or microbial associates on their development, behavior, survival, etc. These efforts could be of huge importance towards the successful management of the red imported fire ants (RIFA). Insect-associated microorganisms have widely demonstrated the capacity to infect their hosts. Notably, fungi, bacteria, viruses, or virus-like organisms have been reported to cause visible infections in RIFA [8,9].

Baseline analysis of fire ants mounds and plant debris would reveal unique or distinct naturally occurring fungal associates of RIFA. As the microbial species within a microbial population may be beneficial or pathogenic to the hosts, isolation and identification of the associated microbes could help identify potential biological control options for these noxious pests. Arguably, more research works should focus on the diversity of fungi associated with *S. invicta*, as the currently available data are limited. Tellingly, a previous study suggested RIFA mound soils as a more desirable source of soil-inhabiting fungi. The collected mound soils exhibited a significantly higher abundance of fungi, where roughly 19 times more colony-forming units than the non-mound soils were recorded [10]. However, most previous studies have focused on investigating various entomopathogenic fungal species as potential biocontrol agents for RIFA, while only few studies have conducted surveys to explore the associated microorganisms of RIFA [11]. Survey studies on RIFA microbial associates conducted in China are still limited, while few studies conducted in the United States and some other countries are available [7,10,12,13,14,15,16,17].

The extraction of novel fungal isolates from different soils and other environmental samples has been accomplished using multiple isolation methods. Fungi isolation involving the use of selective media methods is apparently the most common. Meanwhile, several insect-baiting methods have also been widely reported as effective for the isolation of entomopathogenic fungal species [4,18,19,20,21,22]. Some of these aforementioned studies as well as few other RIFA-related studies have revealed that several generalists and entomopathogenic fungi occur in fire ants mounds [7,10,12,13]. However, Woolfolk et al. [14] argued that the data available from most of these studies are somewhat restricted to only a few locations, therefore making the identification of generalist and entomopathogenic fungal associates of the fire ants still relatively unclear. This has therefore raised the need for expanding the available data on fire ants mounds associated fungal microbes. In this vein, an extensive survey of the red imported fire ants’ associated fungal microbes was conducted in the current study. The current study assessed the species richness, diversity, and densities of the culturable fungi associated with RIFA, plant debris within mounds, and mound soils collected from various cities across Guangdong province of China. It is a general opinion that isolating and accurately identifying the RIFA microbial associates would be vital for the effective management of the red imported fire ants.

## 2. Materials and Methods

### 2.1. Soil and Ants Sampling

**Figure 1:**
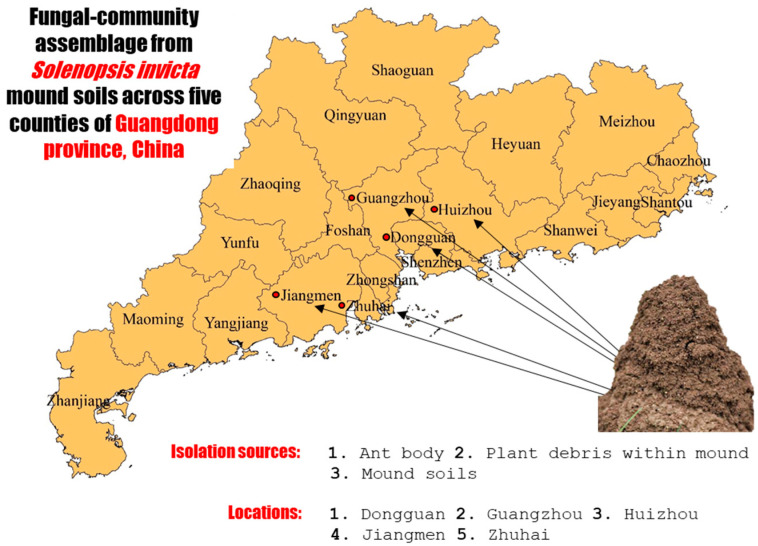
Diagrammatical representation of experimental layout, detailing the various sources of isolation and sampling locations for baseline analysis of fungal associates of RIFA.

During the spring of 2021, soil samples were collected from fire ant mounds from selected locations across five cities, namely Dongguan, Guangzhou, Huizhou, Jiangmen, and Zhuhai, located within Guangdong Province of China (Figure 1).

From each of the five cities, soil samples were collected from three randomly selected mounds. For individual sampling, approximately 500 g of soil was collected from each mound, taken at about 10–15 cm below the surface using a sterilized hand shovel. The soil samples were transported to the laboratory for analysis in sealed plastic bags.

### 2.2. Procedure for Fungi Isolation from Samples

Media preparation: The selective media method was deployed for the isolation of fungal isolates from collected samples. First, 40.1 g of potato dextrose agar (PDA; Guangdong Huankai Microbial Sci. and Tech. Co., Ltd., Guangzhou, China) was dissolved in 1 L of distilled water and was amended with 100 mg/L tetracycline hydrochloride (Sangon) and 300 mg/L streptomycin sulfate (Sangon) to inhibit bacteria growth.

Isolation from soil samples: Prior to microbial isolation, existing clumps and stones were carefully removed from soils using a 2 mm pore sieve. Following the procedure of Dhar et al. [23], about 50 g of soil per individual mound was suspended in 500 mL sterile distilled water and vortexed at 200 rpm for approximately 25–30 min on a rotary shaker at room temperature, enabling the fungal spores present in the soil to be dislodged. This procedure was followed by allowing the soil particles to settle for about 15–20 min, and from the third serial dilution of the supernatant, 100 μL of solution was evenly spread on PDA solid media using a sterile disposable cell spreader. Inoculated plates were transferred into a BOD incubator (BS-1E, China) and incubated at 25 °C for 5 days.

Isolation from mound plant debris: Plant debris were removed from soil, and remnant soils were carefully removed using a brush. The remaining soil clogs were removed from the plant tissues by washing them in sterile distilled water for approximately 1 min. The procedures described by Woolfolk et al. [14] were followed for fungi isolation from plant samples. Plates were supplemented with antibiotics to minimize contamination and incubated for 5 days, as previously described.

Isolation from ant bodies: The isolation procedure was carried out following the guidelines of Baird et al. [7] with slight modifications. In the current study, 24 ants were selected per mound, while each individual media plate received 4 ants (a total of 6 plates per mound). Plates were also supplemented with antibiotics and incubated under similar conditions as previously described. All fungal mycelium emerging from the ant tissues were subsequently sub-cultured in fresh growth medium for up to four weeks until monocultures for all fungal isolates were cultivated.

### 2.3. Morphological Characterization

Following multiple sub-culturing, the monocultures were identified on the basis of their phenotypic distinctiveness prior to phylogenetic characterization. Morphological characterization of fungal strains followed the protocols as described by Humber [24]. Further characterization was performed following the guidelines of Meyer et al. [25], where an optical microscope system equipped with a digital camera was used to analyze the mycelia, conidia, and sporulation structures of individual fungal isolates.

### 2.4. Molecular Identification

Extraction of genomic DNA from fungal cultures (about 7 days old) was completed with the help of genomic DNA extraction kits (Rapid Fungi Genomic DNA Isolation Kit) provided by the manufacturer (Sangon, Shanghai, China). Extracted DNA was amplified using targeted regions specific primers as follows (Table 1).

The PCR procedure includes a 50 μL reaction mix containing 25.0 μL of 2× High-Fidelity PCR MasterMix (Tiangen Biotech, Shanghai, China), 2.0 μL of each primer pairs, 3.0 μL of DNA template, and 18.0 μL PCR-grade water. The PCR conditions set for fungal DNA amplification was strictly in accordance to the guidelines of the manufacturer. Visualization of amplified DNA was completed on 1.0% m/v agarose gel, and Sanger sequencing was conducted by Sangon Biotech Co. Ltd. Guangzhou, China.

### 2.5. Phylogenetic Analysis

BioEdit v 7.1.9. [26] was utilized to manually edit the obtained fungal sequence traces, while multiple loci were edited and aligned using Clustal W [27]. With the help of BLASTn, the reference sequences were downloaded from the *GenBank* database of the National Center for Biotechnology Information (NCBI) (http://www.ncbi.nlm.nih.gov/—accessed on 25 February 2023). In addition, we performed phylogenetic analysis using the sequences produced for the present study and the reference sequences. Neighbor-joining method was deployed for the analysis of sequences based on the maximum composite likelihood via MEGA v. 11.

### 2.6. Statistical Analysis

Using the frequencies of isolation, the biodiversity indices of fungal samples were determined in accordance with a few of the previously described procedures [7,28,29]. Statistical analysis was performed to determine index of diversity (H’), species richness (n), and evenness (J’). Consequently, diversity of fungal species was calculated via Shannon–Wiener index (H’), which was computed as follows: H’ = ∑ PiInPi, Pi = Ni/Nt, where Ni denotes the value for isolates that belong to the i-th genus, while Nt on the other hand represents the value for isolates in the group of interest (i.e., sampling sites or substrates). In addition, the community coefficient (CC) was computed using the following formulae: CC = C/(S_1 + S_2 − C). Here, C represents the value of unique fungal species that is common to both substrates/location under study, while S_1 and S_2 denote the values for fungal species in individual community, i.e., substrate/location 1 and substrate/location 2, respectively.

When required, one-way analysis of variance (ANOVA) was used for data analysis. The least-significance difference (LSD) test at *p* < 0.05 was employed to perform multiple comparison among treatment means.

## 3. Results

### 3.1. Morphological Characterization of Fungal Isolates

**Figure 2:**
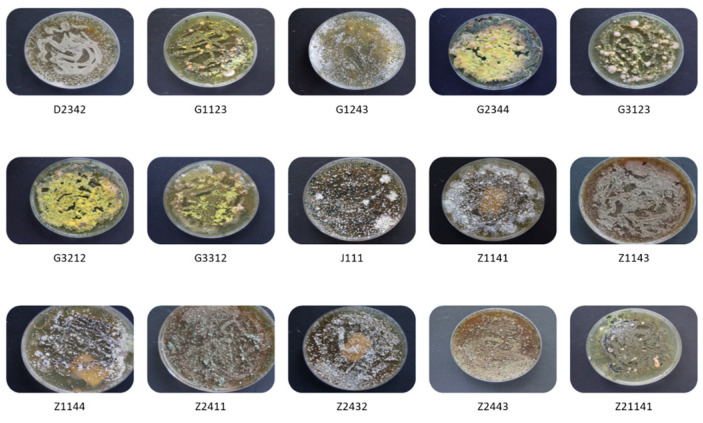
Representative samples of the purified fungal isolates extracted from fire ants, mound soils, and plant debris. Samples were collected across five different cities in Guangdong Province, China.

Following multiple sub-culturing until unique cultures were cultivated for all fungal isolates, morphological identification was conducted to determine their distinctive phenotypic features (Figure 2).

### 3.2. Molecular Identification and Phylogenetic Placement of Isolates

**Figure 3:**
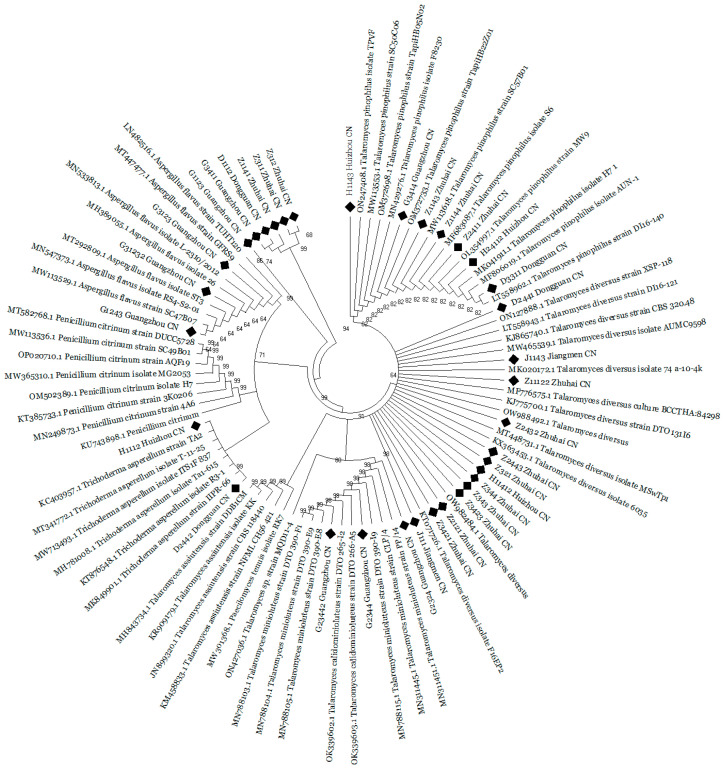
Phylogenetic analysis based on a combined dataset sequence of ITS, 18s, and 28s partial sequences of the characterized fungal isolates. Neighbor-joining method was utilized to infer the evolutionary history, while the evolutionary distances were computed using maximum composite likelihood method. The optimal tree is shown. This analysis involved 92 nucleotide sequences and 273 positions in the final dataset. The fungal taxa used in this study are highlighted with black dots. Evolutionary analyses were conducted in MEGA11.

In addition to morphological characterization, the successful classification of isolates into specific fungal taxa was achieved by molecular identification based on DNA sequences of multiple loci and phylogenetic characterization. In total, 34 fungal taxa were identified, while 12 isolates are yet to be identified. Phylogenetic characterization was carried out using the combined dataset of three loci (SSU + LSU + ITS) while obtaining supplementary sequences available in the database of NCBI (Figure 3). The DNA sequences derived in the current study can be found in *GenBank*, and the accession numbers are provided (Table 2).

### 3.3. Assessment of Fungal Species Richness, Diversity, and Densities

**Figure 4:**
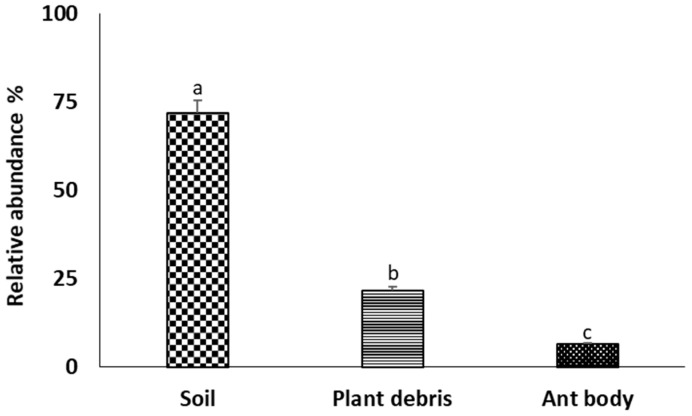
The distribution of fungal taxa across substrates: ant body, plant debris, and mound soils. Bars (±SE) with different letters designate significant differences at *p* < 0.05 (LSD after one-way ANOVA.

For the assessment of fungal species richness, diversity, and densities, fungal isolates were classified into two sub-groups, i.e., sampling sources and locations. In general, the isolation percentage across the fungal taxa varied from 3.9% to 26.5%, and about 24.1% were unidentified. Across various cities and substrates, *T. diversus* was the most commonly extracted taxa at 26.5%. Other taxa with the greatest percent isolation frequencies include *T. pinophilus* (12.8%), *T. minioluteus* (8.3%), and *A. flavus* (6.4%). The identified taxa were unevenly distributed across the three examined environmental samples: soil (71.7%), plant debris (21.7%), and ant bodies (6.5%) (Figure 4).

**Figure 5:**
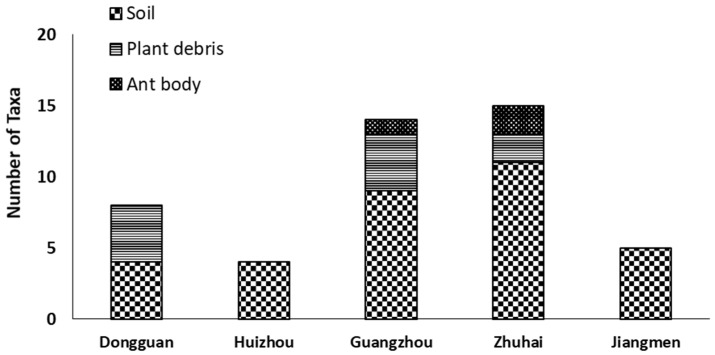
Endophytic fungal taxa cultivated from different substrates collected from five different cities of Guangdong Province, China.

Among the fungal species, only *T. diversus* (62.5%) and *T. pinophilus* (37.5%) were successfully isolated from the ant body, while the predominant fungal species across plant debris was *T. diversus*. All fungal species were successfully isolated from soil samples, where *A. flavus* and *T. minioluteus* were only obtained from soils and not from any other substrates. Only *T. diversus* and *T. pinophilus* were recorded across all substrates. Following the analysis of sampling locations and environmental samples, the results show that the highest taxa abundance values were from mound soils (33) and Zhuhai (15), respectively (Figure 5).

For diversity of species assessment using Shannon’s diversity index, the values for fungal species diversity were 1.49 (H’) and 0.75 (H’) across cities and substrates, respectively. On the other hand, the evenness of fungal species was 0.92 (J’) and 0.68 (J’) across cities and substrates, respectively (Table 3). With regards to the coefficient of community values obtained for fungal species across five different locations and the three substrates examined, values ranged from 0.29 to 0.57 (Table 4). For locations such as Zhuhai, Dongguan, and Huizhou, computed CC values for all paired substrates, namely ant body vs. plant debris, ant body vs. mound soil, or plant debris vs. mound soil, were 0.0, as the fungal species recorded across different substrates were not similar. For other locations, namely Jiangmen and Guangzhou, CC values ranged from 0.33 to 1.0 and 0.00 to 0.33, respectively (Table 5).

## 4. Discussion

The current study conducted a baseline analysis of fungal community assemblage in RIFA mounds across five cities located within Guangdong Province, China. The quest to expand the available data on potential biological control agents of RIFA has been one of the major motivation for conducting scientific studies related to insect-hosts-associated microbe interactions. The diversity, richness, and densities of fungal associates of RIFA mounds were examined across ant body, mound soils, and plant debris within the mounds.

The results revealed unevenness in the distribution of fungal species within substrates and across various locations examined. Among the fungal species, the highest species richness values and abundance (total isolations) were recorded in the mound soils, while the rate was lower for ant body and mound plant debris. With regards to the isolation sites, the highest fungal species richness value was recorded in the samples collected from Dongguan and Guangzhou, while the fungal taxa abundance level was highest in the samples collected from Zhuhai.

The overall high value of species richness from soil samples was similar to the study of Woolfolk et al. [14], where evidence of fungal species richness in mound soils at a significantly higher level than in plant debris within the mound and ant body was provided. Similarly, another study conducted by Baird et al. [7] also reported the highest total species richness values in the mound soils, which was significantly higher than values recorded for ant bodies and plant debris within mound soils.

The current study reveals a successful isolation of some beneficial ant–fungal associates, where the most commonly isolated taxa across all substrates and locations are *T. diversus*, *T. pinophilus*, *A. flavus*, and *T. asperellum*. Most of the identified fungal species could be classified as generalists, while only a few have previously been reported as insect pathogenic fungi. For instance, *A. flavus* in *Aphis fabae* [30], *P. citrinum* in *Spodoptera frugiperda* [31], and *T. asperellum* [32]. The possibility of some species of entomopathogenic fungi existing within the RIFA mound has been reported. For instance, in a related study, the cosmopolitan insect pathogenic fungus *Beauveria bassiana* (Balsamo) Vuillemin was successfully isolated from mound soils, plant debris within mound soils, and ant bodies [7]. Similarly, two other common insect pathogenic fungi, i.e., *Purpureocillium lilacinum* and *Metarhizium anisopliae* (Metschnikoff) Sorokin, were isolated from mound soils, mounds plant debris, and ant bodies by Woolfolk et al. [14]. These two studies are similar to several other related studies where a larger percentage of the cultured fungi belong to the artificial assemblage fungi imperfecti (Deuteromycetes), while the majority were documented as non-pathogenic fungal species [10,13,33].

The findings of the present study are similar to numerous other studies that have revealed the possibility of fire ant mounds serving as a good source of important or beneficial microbial symbionts of RIFA [7,14,34]. However, the specific roles played by the fungal species characterized in the current study in RIFA populations regulating and other environmental processes are yet to be clearly defined. For example, the definite interactions between RIFA and *T. diversus*, which was the most abundant species across all examined substrates and locations, have not been adequately documented in the literature, whereas a number of fungal species that exist as microbial associates of ant mounds have been reported with the potential of naturally regulating colony populations. This possibility was reported in *Paecilomyces lilacinus* [7]. This fungus draws advantage from its ability to survive in a wide range of agricultural ecosystems as a saprophyte, entomopathogen, as well as nematophagous [35]. Similarly, a number of *Fusarium* species are also known for their saprophytic lifestyles, where they can exist as opportunists on plant debris within mound soils or as plant parasites in many living plant species, although their effects or benefits on *S*. *invicta* or their mounds have not been well documented [14]. As fungal species are able to survive in various habitats, their existence in fire ant mounds has been suggested to be secondary [7]. This is similar to many fungal species that have been classified as rhizosphere-competent, hence their ability to colonize and survive in variety of soils across different regions [19]. It has also been found that certain fungal associates of the RIFA could display a host-protective mechanism against foreign insect pathogenic fungi. This was evident in RIFA colonies’ association with *Hypocrea lixii*, which appears to protect the ants colonies from being colonized by other entomopathogenic fungi such as *B. bassiana* or *M*. *anisopliae* [14].

Most of the fungal species classified in the current study have previously been isolated from various soil types (including mound and non-mound soils and cultivated and non-agricultural soils), regions, or habitats (including agricultural and forest systems). Moreover, a few species have been reported as phytopathogens of cultivated crops in many agricultural ecosystems. For instance, *P. citrinum* and *A. flavus* have both been reported as saprophytes with the ability to colonize plant debris within the soil as well as existing as plant parasites, serving as causal organisms of several plant infections [36,37,38,39]. Although the cultured fungal species have demonstrated the ability to survive in different soil types and habitats., some previous studies have provided evidence of ant mound soils with greater abundance or richness in fungal species in comparison to non-mound soils. For instance, Zettler et al. [10] found about 19 times more colony-forming units in mound soils, although lower fungal species richness and diversity was reported in the mound soils. On the other hand, Woolfolk et al. [14] suggested that the habitat or geographical location of the isolation sites could exert much more influence on fungal species abundance than the ant colonies. In addition, temperature, PH, rainfall, and several other environmental conditions have been reported to greatly affect fungal diversity within RIFA mounds [10].

## 5. Conclusions

The current findings reveal the existence of diverse fungal species within the RIFA mound soils, plant remnants deposited within the mound, and the body of ants. The identified fungal species were found to be unevenly distributed across the substrates and locations examined. Several entomopathogenic fungal species such as *B*. *bassiana*, *M*. *anisopliae*, and *P. lilacinum* have been found existing within the RIFA mounds, where they could play the role of colony population regulators by nature. However, none of these three insect pathogenic species was successfully recovered in the current study. Notably, few of the species recorded in this study have been reported as insect pathogenic fungi in some insects. The specific roles played by the cultured fungal species in RIFA survival, growth, invasion, and other ecological functions are still relatively unknown. Future research should be focused in this direction.

## Figures and Tables

**Table 1:** List of primer pairs selected for fungal DNA fragments amplification.

Targeted Region	Primer Used	
Internal transcribed spacers region	ITS 1	5′-TCCGTAGGTGAACCTGCGG -3′
	ITS 4	5′-TCCTCCGCTTATTGATATGC-3′
Partial 18S rRNA (SSU)	NS1	5′-GTAGTCATATGCTTGTCTC-3′
	NS4	5′-CTTCCGTCAATTCCTTTAAG-3′
Partial 28S rRNA (LSU)	LR0R	5′-ACCCGCTGAACTTAAGC-3′
	LR5	5′-TCCTGAGGGAAACTTCG-3′

**Table 2:** Fungal taxa characterized from different substrates collected from five cities of Guangdong Province of China.

Isolate Number	Taxa	Location of Isolation	Source of Isolation	*GenBank* Accession Number		
				ITS	SSU	LSU
D1112	*Penicillium citrinum*	Dongguan	MS	OQ518404	OQ518708	OQ538334
D1314	Unidentified fungal sp.	Dongguan	MS	-	-	-
D2133	Unidentified fungal sp.	Dongguan	MS	-	-	-
D2342	Unidentified fungal sp.	Dongguan	MS	-	-	-
D23422	Unidentified fungal sp.	Dongguan	PD	-	-	-
D2441	*Talaromyces diversus*	Dongguan	PD	OQ518374	OQ518686	OQ538307
D2442	*Trichoderma asperellum*	Dongguan	PD	OQ518375	OQ518687	OQ538308
D3311	*Talaromyces pinophilus*	Dongguan	PD	OQ518405	OQ518709	OQ538335
G1123	*Talaromyces pinophilus*	Guangzhou	MS	OQ518376	OQ518688	OQ538309
G1233	Unidentified fungal sp.	Guangzhou	MS	-	-	-
G12332	Unidentified fungal sp.	Guangzhou	MS	-	-	-
G1243	*Aspergillus flavus*	Guangzhou	MS	OQ518377	OQ518689	OQ538310
G2324	*Talaromyces minioluteus*	Guangzhou	MS	OQ518378	-	-
G2344	*Talaromyces minioluteus*	Guangzhou	MS	OQ518379	OQ518690	OQ538311
G23442	*Talaromyces minioluteus*	Guangzhou	MS	OQ518399	-	-
G3123	*Aspergillus flavus*	Guangzhou	MS	OQ518380	OQ518691	OQ538312
G31232	*Aspergillus flavus*	Guangzhou	MS	OQ518400	OQ518706	OQ538330
G3212	Unidentified fungal sp.	Guangzhou	PD	-	-	-
G32122	Unidentified fungal sp.	Guangzhou	PD	-	-	-
G3312	Unidentified fungal sp.	Guangzhou	PD	-	-	OQ538313
G3411	*Penicillium citrinum*	Guangzhou	PD	OQ518381	OQ518692	OQ538314
G3414	*Talaromyces pinophilus*	Guangzhou	AB	OQ518382	OQ518693	OQ538315
H1112	*Trichoderma asperellum*	Huizhou	MS	OQ518401	-	OQ538331
H11412	*Talaromyces diversus*	Huizhou	MS	OQ518402	OQ518707	OQ538332
H1143	*Talaromyces pinophilus*	Huizhou	MS	OQ518373	OQ518685	OQ538306
H24112	*Talaromyces pinophilus*	Huizhou	MS	OQ518403	-	OQ538333
J111	*Trichoderma asperellum*	Jiangmen	MS	-	OQ518694	OQ538316
J1143	*Talaromyces diversus*	Jiangmen	MS	OQ518383	-	OQ538317
J11432	Unidentified fungal sp.	Jiangmen	MS	-	-	-
J2133	Unidentified fungal sp.	Jiangmen	MS	-	-	-
J2143	Unidentified fungal sp.	Jiangmen	MS	-	-	-
Z11122	*Talaromyces diversus*	Zhuhai	MS	OQ518384	OQ518695	OQ538318
Z1141	*Talaromyces diversus*	Zhuhai	MS	OQ518385	-	OQ538319
Z1143	*Talaromyces pinophilus*	Zhuhai	MS	OQ518386	-	OQ538320
Z1144	*Talaromyces pinophilus*	Zhuhai	MS	OQ518387	OQ518696	OQ538321
Z2121	*Talaromyces* sp.	Zhuhai	MS	OQ518388	OQ518697	OQ538322
Z2411	*Talaromyces* sp.	Zhuhai	MS	OQ518389	OQ518698	OQ538323
Z2432	*Talaromyces diversus*	Zhuhai	MS	OQ518390	OQ518699	OQ538324
Z2443	*Talaromyces diversus*	Zhuhai	MS	OQ518391	-	-
Z311	*Talaromyces* sp.	Zhuhai	MS	OQ518392	OQ518700	OQ538325
Z312	*Talaromyces* sp.	Zhuhai	MS	OQ518393	OQ518701	OQ538326
Z321	*Talaromyces diversus*	Zhuhai	MS	OQ518394	-	OQ538327
Z3421	*Talaromyces diversus*	Zhuhai	PD	OQ518395	OQ518702	OQ538328
Z3423	*Talaromyces diversus*	Zhuhai	PD	OQ518396	OQ518703	OQ538329
Z343	*Talaromyces diversus*	Zhuhai	AB	OQ518397	OQ518704	-
Z344	*Talaromyces diversus*	Zhuhai	AB	OQ518398	OQ518705	-

MS, mound soil; AB, ant body; PD, plant debris within mounds.

**Table 3:** Fungal taxa abundance, evenness, richness of species, and diversity index across sampling locations and substrates.

**Location**	**Dongguan**	**Huizhou**	**Guangzhou**	**Zhuhai**	**Jiangmen**	**H’**	**J’**
	**17.4 (4)**	**8.7 (3)**	**30.4 (4)**	**32.6 (3)**	**10.7 (3)**	**1.49**	**0.92**
**Substrates**	Soil	Plant debris	Ant body			**H’**	**J’**
	71.7 (7)	21.7 (4)	6.5 (2)			**0.75**	**0.68**

The values presented under each substrate or sampling location represent the total number (percentage) of distinct fungal taxa, whereas the numbers in parentheses refer to richness of species (S). The values under H’ denote Shannon index of diversity, while evenness of species is designated as J’ across sampling locations or substrates, respectively.

**Table 4:** The community coefficient (CC) of fungal taxa cultivated from different substrates.

Substrates	CC
AB–MS	0.29
AB–PD	0.50
PD–MS	0.57

**Table 5:** The community coefficient (CC) of fungal taxa cultivated from different substrates within the individual sampling location.

Location	Substrates	CC
Dongguan	AB–PD	0.0
	AB–MS	0.0
	PD–MS	0.0
Guangzhou	AB–PD	0.00
	AB–MS	0.33
	PD–MS	0.0
Huizhou	AB–PD	0.0
	AB–MS	0.0
	PD–MS	0.0
Jiangmen	AB–PD	1.00
	AB–MS	0.33
	PD–MS	0.33
Zhuhai	AB–PD	0.0
	AB–MS	0.0
	PD–MS	0.0

MS, mound soil; AB, ant body; PD, plant debris within mounds.

## Data Availability

Data supporting the results can be found in NCBI’s *GenBank* database following this link—http://www.ncbi.nlm.nih.gov/—accessed on 25 February 2023.
